# Efficacy and safety of once-weekly insulin icodec compared to once-daily insulin g U-100 in patients with type II diabetes: a systematic review and meta-analysis

**DOI:** 10.1186/s13098-024-01305-z

**Published:** 2024-04-03

**Authors:** Syed Zia Saleem, Areeba Fareed, Syed Muhammad Muneeb Akhtar, Solay Farhat, Amira Mohamed Taha, Aymar Akilimali

**Affiliations:** 1 Department of Medicine, Dow University of Health Sciences, Karachi, Pakistan; 2 Department of Medicine, Karachi Medical and Dental College, Karachi, Pakistan; 3 Faculty of Science, Lebanese University, Beirut, Lebanon; 4 Faculty of Medicine, Fayoum University, Fayoum, Egypt; 5 Department of Research, Medical Research Circle, Bukavu, DR Congo

**Keywords:** Insulin Icodec, Insulin Glargine U-100, Type 2 diabetes (T2D), Once-weekly Insulin regimen, Glycemic control

## Abstract

**Background//Objective:**

Diabetes affects millions of people globally, despite treatment options, adherence and other factors pose obstacles. Once-weekly Insulin Icodec, a novel basal Insulin analog with a week-long half-life, offers potential benefits, enhancing convenience, adherence, and quality of life for improved glycemic control. This systematic review and meta-analysis aimed to assess the efficacy and safety of once-weekly Insulin Icodec compared to once-daily Insulin Glargine U-100 in individuals with type II diabetes (T2D).

**Methods:**

A comprehensive literature search was conducted using PubMed, and Cochrane Library databases before September 2023 to identify relevant Randomized control trials (RCTs) with no language restrictions following PRISMA guidelines. The Cochrane risk-of-bias tool was used for quality assessment. All statistical analyses were conducted using RevMan (version 5.4; Copenhagen: The Nordic Cochrane Centre, The Cochrane Collaboration, 2014).

**Result:**

Four RCTs published from 2020 to 2023 with a cumulative sample size of 1035 were included. The pooled mean difference (MD) revealed a 4.68% longer TIR (%) with Insulin Icodec compared to Insulin Glargine U-100 [{95% CI (0.69, 8.68), p = 0.02}], the estimated mean changes in HbA1c (%) and FPG (mg%) were found to be insignificant between the two groups [MD = − 0.12 {95% CI (− 0.26, 0.01), p = 0.07}] and [MD = − 2.59 {95% CI (− 6.95, 1.78), p = 0.25}], respectively. The overall OR for hypoglycemia was also nonsignificant between the two regimens 1.04 [{95% CI (0.71, 1.52), p = 0.84}]. Other safety parameters were similar between the two groups.

**Conclusions:**

Switching from daily Insulin Glargine U-100 to weekly Insulin Icodec showed longer TIR (%) as well as similar blood glycemic control and safety profile. Hence, it may be a good alternate option for management of longstanding T2D.

**Supplementary Information:**

The online version contains supplementary material available at 10.1186/s13098-024-01305-z.

## Introduction

Diabetes is a chronic and progressive illness that demands multiple interventions to reduce its burden. Type 2 diabetes (T2D) is expected to affect 6.28% (462 million) of the world’s population [1] and estimated to affect more than 1.3 billion people worldwide in the next 30 years [2]. Despite the availability of various treatment options, achieving adequate glycemic control remains challenging for many patients due to multiple factors, including poor adherence, fear of injections, hypoglycemia, weight gain, and treatment costs [3–7]. Once-daily basal Insulin analogs have partially addressed these concerns, but research indicates that patients would value further dosing frequency reduction to once weekly. Once-weekly Insulin therapy may improve convenience, adherence, and quality of life, potentially leading to better glycemic control [8]. To address this issue, once-weekly Insulin Icodec, a novel basal Insulin analog with a half-life of approximately one week, has been developed.

When transitioning from a daily basal Insulin regimen to once weekly Icodec, a supplemental dose (loading dose) may be necessary during the initial weeks to maintain glycemic control until a steady state is achieved. Insulin Icodec has a stable pharmacokinetic and pharmacodynamic profile, allowing for once-weekly dosing [9]. Its long half-life is attributed to strong, reversible albumin binding, reduced enzymatic degradation, and slow receptor-mediated clearance. Upon injection, Icodec forms an inactive depot bound to albumin, providing a continuous release throughout the week [10]. Its extended half-life and once-weekly dosing regimen offer advantages in terms of patient adherence and quality of life.

Previous meta-analyses [11, 12] have compared this novel weekly Insulin regimen with either once daily Insulin Glargine U-100 or degludec concluding similar glycemic efficacy coupled with better or similar safety profiles. However, further research has been released since then, hence, we sought to analyze the comparison in the light of the most recent evidence with a larger sample size. This updated systematic review and meta-analysis assessed the efficacy and safety of once-weekly Insulin Icodec compared to once daily Insulin Glargine U-100 in patients with T2D. The trials included participants who were Insulin naive as well as those who were already on basal Insulin treatment for T2D. The primary outcomes analyzed were percentage time in glucose range TIR (%), estimated mean reduction in glycated hemoglobin (HbA1c) (%) and hypoglycemic episodes including alert and combined clinically significant and severe.

## Methods

### Data sources and search

**Fig. 1:**
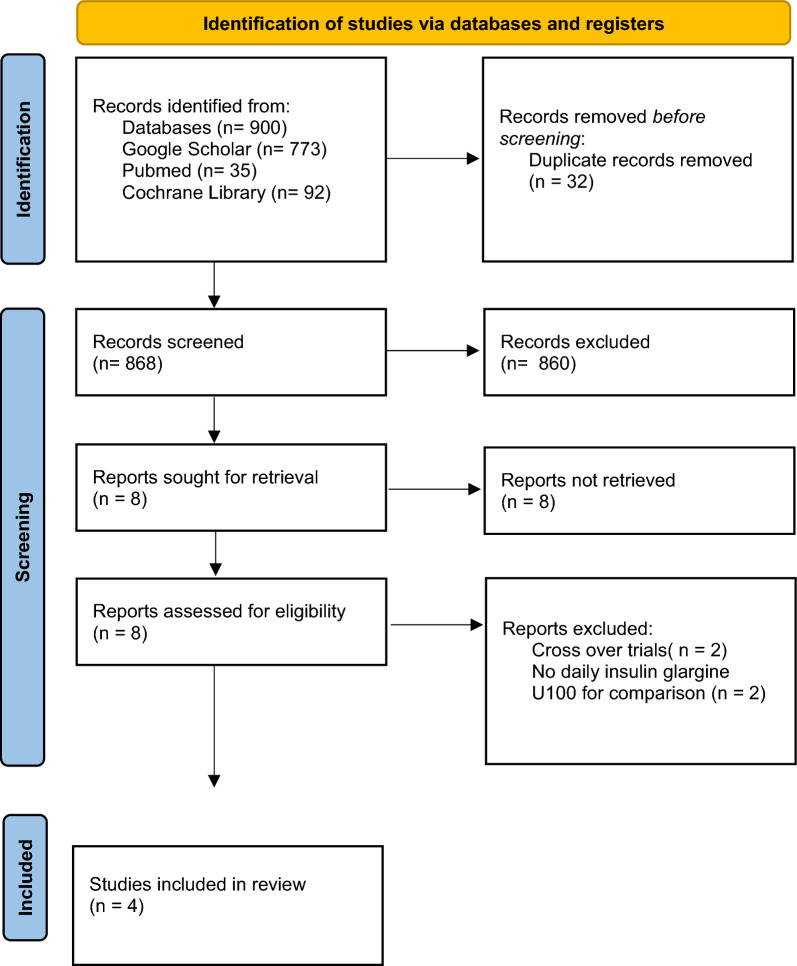
PRISMA Flow Diagram of the Literature Search Process

This study followed the 2020 PRISMA (Preferred Reporting Items for Systematic Review and Meta-Analysis) guidelines [13] as shown in Fig. 1. Our protocol was registered with PROSPERO, The International Prospective Register of Systematic Reviews with registration no. CRD42023472133.

A comprehensive literature search was conducted in the PubMed, and Cochrane Library databases till August 2023 to identify relevant studies. The search string included various combinations of key terms such as “Insulin” Or “Icodec “Insulin Icodec”, “Diabetes”, and “Glargine U-100, articles were retrieved and identified manually for further evaluation. Titles, abstracts, full texts, and reference lists of all identified studies were reviewed. The relevant literature references were carefully checked for potentially eligible studies. No restrictions regarding country, race, or publication language were set. Reference lists from related main studies and review articles were also checked for additional relevant studies.

### Inclusion and exclusion criteria

The inclusion criteria for eligibility were as follows:

#### PICOs

##### Participants

Adult Patients (age 18–72 years, BMI 18.5–37.9 kg/m2, HbA1c % ≤ 75 mmol/mol [≤ 9.0%]) with T2D.

##### Intervention(s) and comparator

Randomized control trials (RCTs) comparing the use of once-weekly Insulin Icodec to once-daily Insulin Glargine.

##### Outcomes

The primary endpoint investigated was estimated mean change in TIR (%), HbA1c (%), with a focus on estimated mean percentage change from baseline and hypoglycemia incidence both alerts and combined clinically significant and severe. Additional assessments included shifts in estimated mean reduction in Fasting plasma blood glucose (FPG) levels (mg/dL), changes in body weight (kg) from baseline, proportion of participants achieving HbA1c % levels lower than 7% and monitoring any adverse events as well as those probably or possibly associated with basal Insulin, injection site reactions, hypersensitivity reactions.

The exclusion criteria included: (a) single-arm studies (b) clinical trials with unavailable results (c) nonrandomized trials, review articles, nonhuman studies, case reports, case series, editorials, abstracts, reviews, comments and letters, expert opinions, studies without original data, and duplicate publications.

### Data extraction

Two investigators (SZS and SMMA) independently extracted the following information from each included study: study characteristics (first author, year of publication, country, sample size, and study type), participant baseline characteristics, and any TEAEs, AEs possibly or probably related to basal Insulin, injection site reaction, hypersensitivity reaction, hypoglycemia alert, clinically significant or severe hypoglycemia, estimated mean difference (MD) in TIR (%), estimated mean HbA1c (%) change from baseline, estimated mean FPG (mg/dL) change from baseline and mean body weight (kg) change from baseline. Any discrepancy between data extractions was resolved by the discussion or consulted by the third author (AF).

### Quality assessment

RCTs were evaluated using the Cochrane Risk of Bias Assessment Tool (ROB1) [14]. Seven components were assessed: (1) random sequence generation, (2) allocation concealment, (3) blinding of participants and personnel, (4) blinding of outcome assessment, (5) incomplete outcome data, (6) selective reporting, and (7) other bias.

### Statistical analysis

RevMan (version 5.4; Copenhagen: The Nordic Cochrane Centre, The Cochrane Collaboration, 2014) along with R Statistical Software [15] and meta package v4.17–0 [16], was used for all statistical analyses. To assess the continuous variables, we calculated the weighted mean difference (MD) and 95% confidence interval (CI). The inverse variance method was used for continuous outcomes. For binary outcomes, the Mantel–Haenszel method was utilized, and we calculated the odds ratio, which measures the ratio of the odds of an event occurring in one group compared to the other. We incorporate the Paule-Mandel estimator for Tau ^^2^ to address potential heterogeneity among the studies in the meta-analysis. By incorporating this estimator, we sought to improve the accuracy and robustness of our pooled effect estimates. This approach allowed us to account for the variability in effect sizes across different studies, leading to more reliable and informative results.

To assess the potential statistical heterogeneity among trials, Higgins I^2^ statistics were used. The I^2^ statistic reveals the percentage of variation between studies owing to heterogeneity rather than chance or sampling error. An outcome of > 75% indicates considerable heterogeneity. When heterogeneity was high, subgroup or sensitivity analysis was used to identify the sources of heterogeneity. Leave-one-out analysis was used to examine the influence of individual studies on the overall pooled effect estimate and it involves iteratively excluding one study at a time and recalculating the effect size. We used a common effect model for the analysis if there was no heterogeneity; otherwise, a random effects model was used. A forest plot was generated to visually display the effect sizes of each study, along with their corresponding confidence intervals. Additionally, the plot showed the overall pooled effect estimate, providing a comprehensive and graphical representation of the meta-analysis results. The p < 0.05 was considered statistically significant.

## Results

### Literature search and study characteristics

A total of 900 studies were identified after the initial search. After removing 32 duplicates, 868 studies were screened, and 860 were excluded based on the titles and abstracts. The full text of the remaining eight studies was reviewed. Ultimately, four studies [17–20] were found to be eligible for inclusion, shown in detail in (Fig. 1), while the other four were excluded (Additional file 1: Table S1).

Among the four included studies, one was published in 2020 [17], two in 2021 [18, 19], and another one in 2023 [20]. All the studies were double-blinded and had parallel group designs, and three were open-label. The main characteristics of the included studies, such as the mean age of the participants in the study and control groups, are presented in (Table 1).

**Table 1:** Study characteristics of the included studies

First author and study year	Study location	Study design	Groups	No. of participants (n)	Sex	Age (Years)	BMI (kg/m^2^)	Diabetes duration, years	HbA1c %, %
Mathieu, C. et al. [20]	Multicenter	Phase 3a, randomized open-label, multicenter	Icodec	291	M = 154 (53%)	59·7 (10·1)	30·5 (5·0)	18·0 (9·1)	8·29 (0·86)
			Glargine U-100	291	M = 150 (52%)	59·9 (9·9)	30·0 (5·0)	16·3 (7·7)	8·31 (0·90)
Lingvay, I et al. [18]	Multicenter	Randomized active-controlled parallel-group multicenter, multi-national open-label, phase 2, treat-to-target trial	Icodec						
			Tit A	51	M = 52.9	59.8 (9.1)	32.3 (4.8)	9.8 (7.2)	8.0 (0.7)
			Tit B	51	M = 54.9	61.2 (8.0)	31.4 (4.7)	9.6 (4.9)	8.1 (0.8)
			Tit C	52	M = 53.8	61.4 (8.0)	30.8 (3.8)	9.2 (4.4)	8.2 (0.9)
			Glargine U-100	51	M = 52.9	60.2 (8.1)	30.6 (4.7)	11.8 (6.8)	8.2 (0.8)
Bajaj, H. S. et al. [19]	Multicenter	Multicenter, open-label, randomized, active-controlled, parallel-group, treat-to-target phase 2 trial	Icodec						
			LD (loading dose)	54	M = 39 (72.2)	62.4 ± 7.2	30.2 ± 4.3	13.8 ± 7.7	7.8 (0.7)
			Glargine U-100	50	M = 33 (66.0)	60.5 ± 7.9	30.3 ± 5.0	14.8 ± 8.1	7.9 (0.7)
Rosenstock, J et al. [17]	Multicenter	Randomized, double-blind double-dummy, phase 2 trial	Icodec	125	M = 70 (56%)	59.7 ± 8.2	31.1 ± 4.9	10.5 ± 8.4	8.09 ± 0.70
			Glargine U-100	122	M = 69 (56.6%)	59.4 ± 9.5	31.4 ± 4.4	8.8 ± 6.1	7.96 ± 0.65

### Quality assessment

**Fig. 2:**
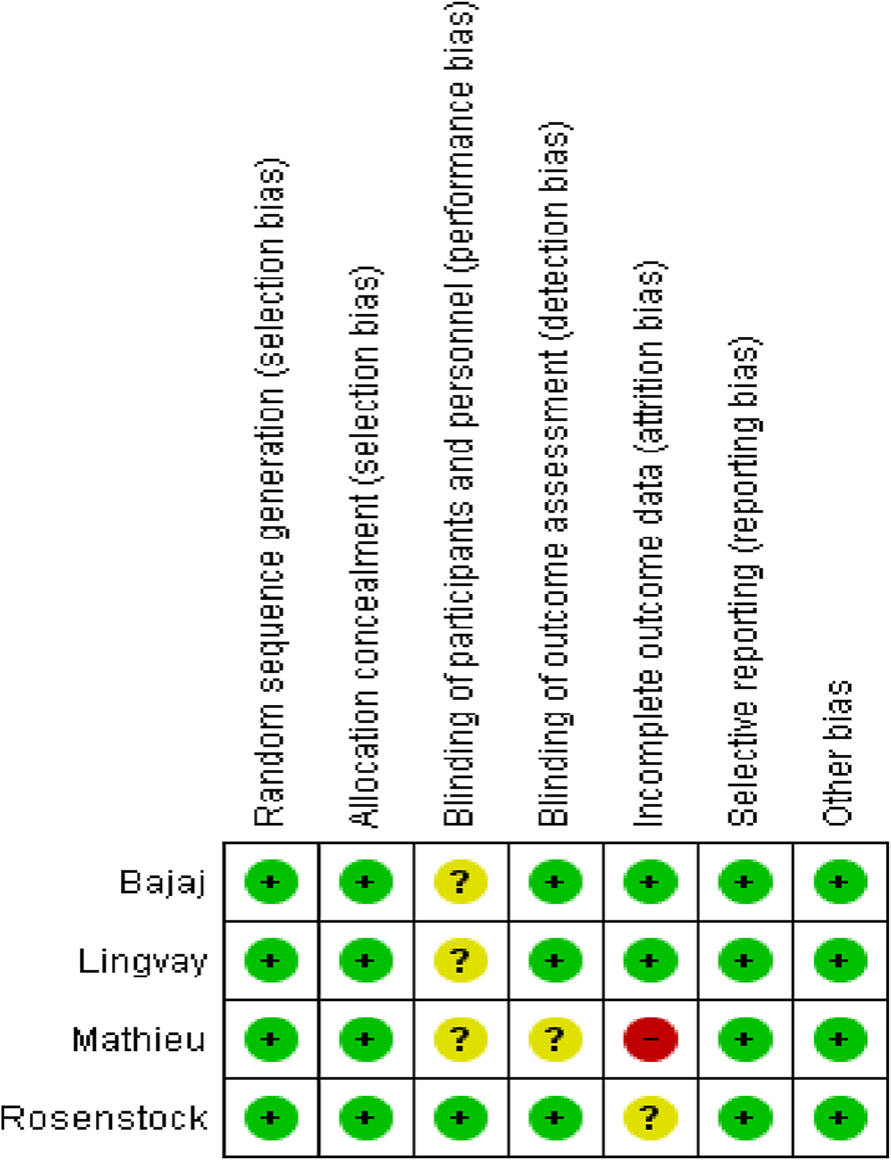
Risk of bias summary. The Cochrane “risk of bias” tool was used for quality assessment. Green for “no risk” and yellow for “unclear risk”

RCTs were evaluated qualitatively using the Cochrane Risk of Bias Assessment Tool (ROB1) [14], findings presented in (Fig. 2). All studies were considered to be of high quality and had low risk of bias. Moreover, publication bias was not assessed for any of the outcomes as the number of included studies did not exceed 10.

### Glycemic parameters

**1. Estimated Mean Change in TIR (%)**

**Fig. 3:**
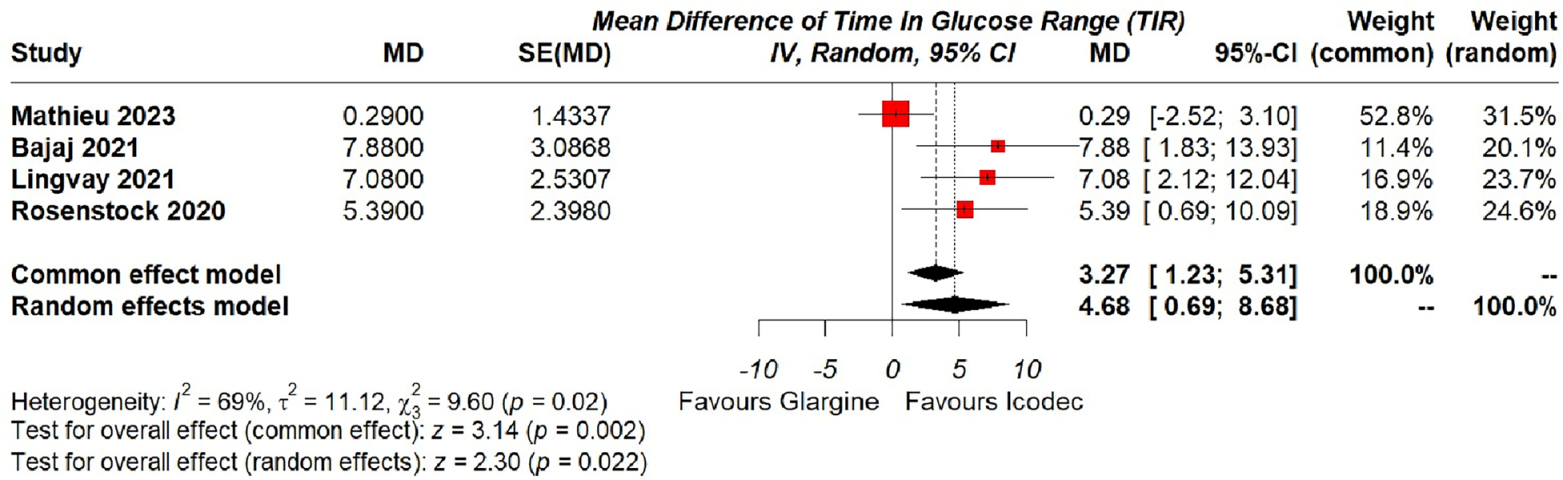
Forest plot of comparison: 1 Once weekly Insulin Icodec vs Once daily Insulin Glargine U-100, outcome: 1.1 Estimated Mean Change in Time with glucose in range (%) from baseline. Pooled analysis has been shown based on both common and random effects model

The pooled analysis included all included studies with a sample size of 1035, random effects model was deemed suitable for this analysis, which demonstrated a significant 4.68% extended TIR with Insulin Icodec as compared to the once daily Glargine {95% CI (0.69, 8.68), p = 0.02, I^2^ = 69%}, shown in (Fig. 3). For the moderate heterogeneity associated with the overall result we performed leave-one-out analysis, removing the outlier study by Mathieu et al. [20], which resulted in complete resolution of heterogeneity and the MD still being significant 6.60 {95% CI (3.63, 9.57), p < 0.001, I^2^ = 0%}, (Additional file 2: Fig. S1). Furthermore, we analyzed the heterogeneity in detail, by employing various estimation methods, all of which resulted in similar I^2^ values, shown in (Additional file 2: Fig. S2).

**2. Estimated Mean Change in HbA1c (%)**

**Fig. 4:**
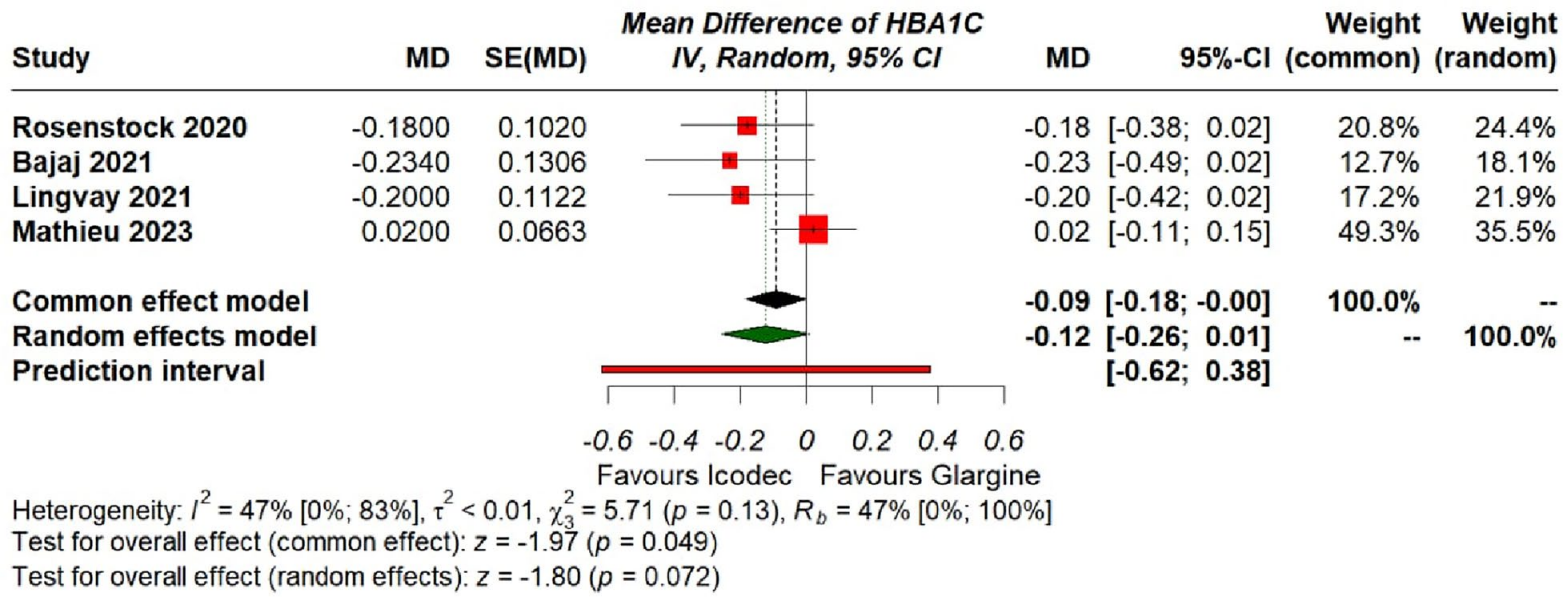
Forest plot of comparison: 1 Once weekly Insulin Icodec vs Once daily Insulin Glargine U-100, outcome: 1.2 Estimated Mean Change in HbA1c (%) from baseline. Pooled analysis has been shown based on both common and random effects model

All the included studies with a combined patient population of 1035 were pooled using a common effects model, which showed significant difference between the two insulins [MD = -0.09 {95% CI (− 0.18, 0.00), p = 0.05, I^2^ = 47%}], presented in (Fig. 4). However, when we applied a random effects model to incorporate the heterogeneity in between studies the pooled result showed an insignificant difference between the two drugs in lowering mean HbA1c (%) from baseline [MD = − 0.12 {95% CI (− 0.26, 0.01), p = 0.07, I^2^ = 47%}), (Fig. 4).

Leave-one-out analysis was conducted to find out the outlier study, after removing the study by Mathieu et al. [20], the heterogeneity reduced to 0% and the overall result turned to significantly better reduction in HbA1c (%) with Insulin Icodec [MD = − 0.20 {95% CI (− 0.33, 0.07), p = 0.002, I^2^ = 0%}], (Additional file 2: Fig. S3).

**3. Estimated Mean Change in FPG (mg/dL)**

**Fig. 5:**
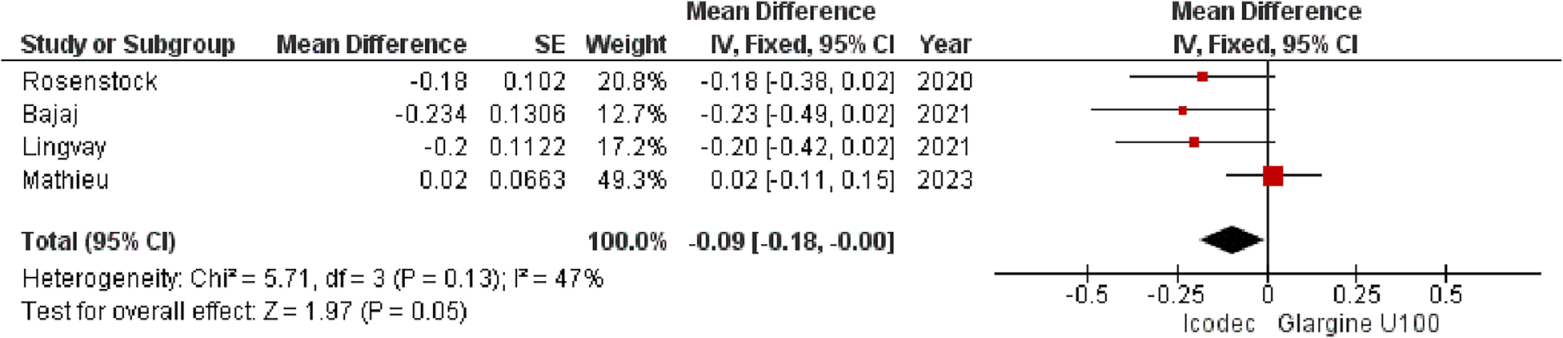
Forest plot of comparison: 1 Once weekly Insulin Icodec vs Once daily Insulin Glargine U-100, outcome: 1.3 Estimated Mean Change in Fasting Plasma Glucose (md/dL) from baseline

All the included studies were pooled for this analysis. No significant difference between Icodec and Once-Daily Insulin Glargine U-100 were shown with a MD of − 2.59 {95% CI (− 6.95, 1.78), p = 0.25, I^2^ = 0%}, shown in (Fig. 5).

**4. HbA1c Lower Than 7%**

**Fig. 6:**
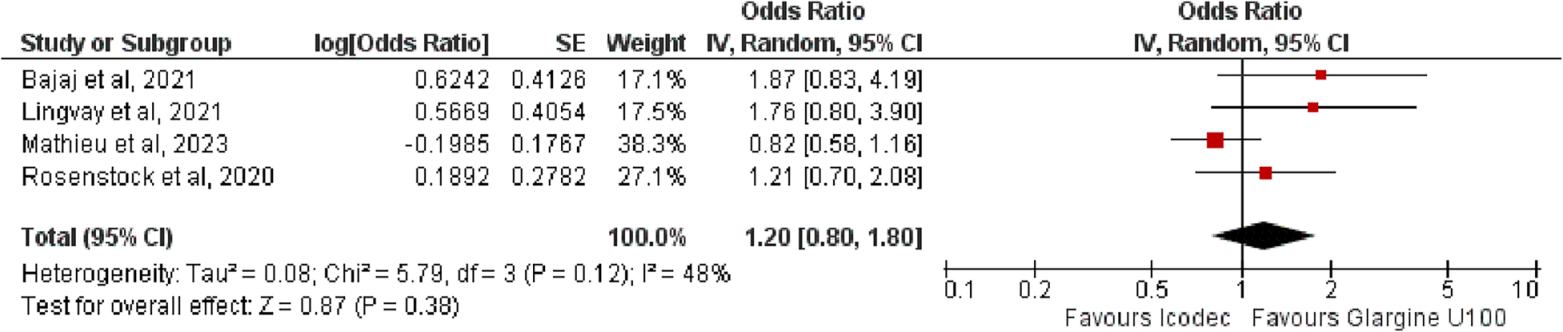
Forest plot of comparison: 1 Once weekly Insulin Icodec vs Once daily Insulin Glargine U-100, outcome: 1.4 Odds ratio of participants achieving HbA1c < 7%

The OR for participants reaching HbA1c lower than 7% was 1.20 {95% CI (0.80, 1.80), p = 0.38, I^2^ = 48%}, showing no significant difference between the two comparators, (Fig. 6).

### Safety parameters

**1. Any Adverse Event**

**Fig. 7:**
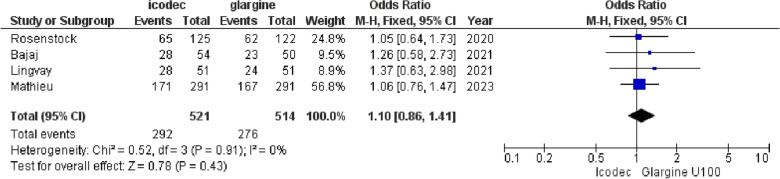
Forest plot of comparison: 1 Once weekly Insulin Icodec vs Once daily Insulin Glargine U-100, outcome: 1.5 Odds ratio for Any Adverse Event

The pooled OR for any adverse event was 1.10 {95% CI (0.86, 1.41), p = 0.43, I^2^ = 0%}, indicating no significant difference between weekly Icodec and Once-Daily Insulin Glargine U-100 with homogenous results, (Fig. 7).

**2. Overall Hypoglycemia**

**Fig. 8:**
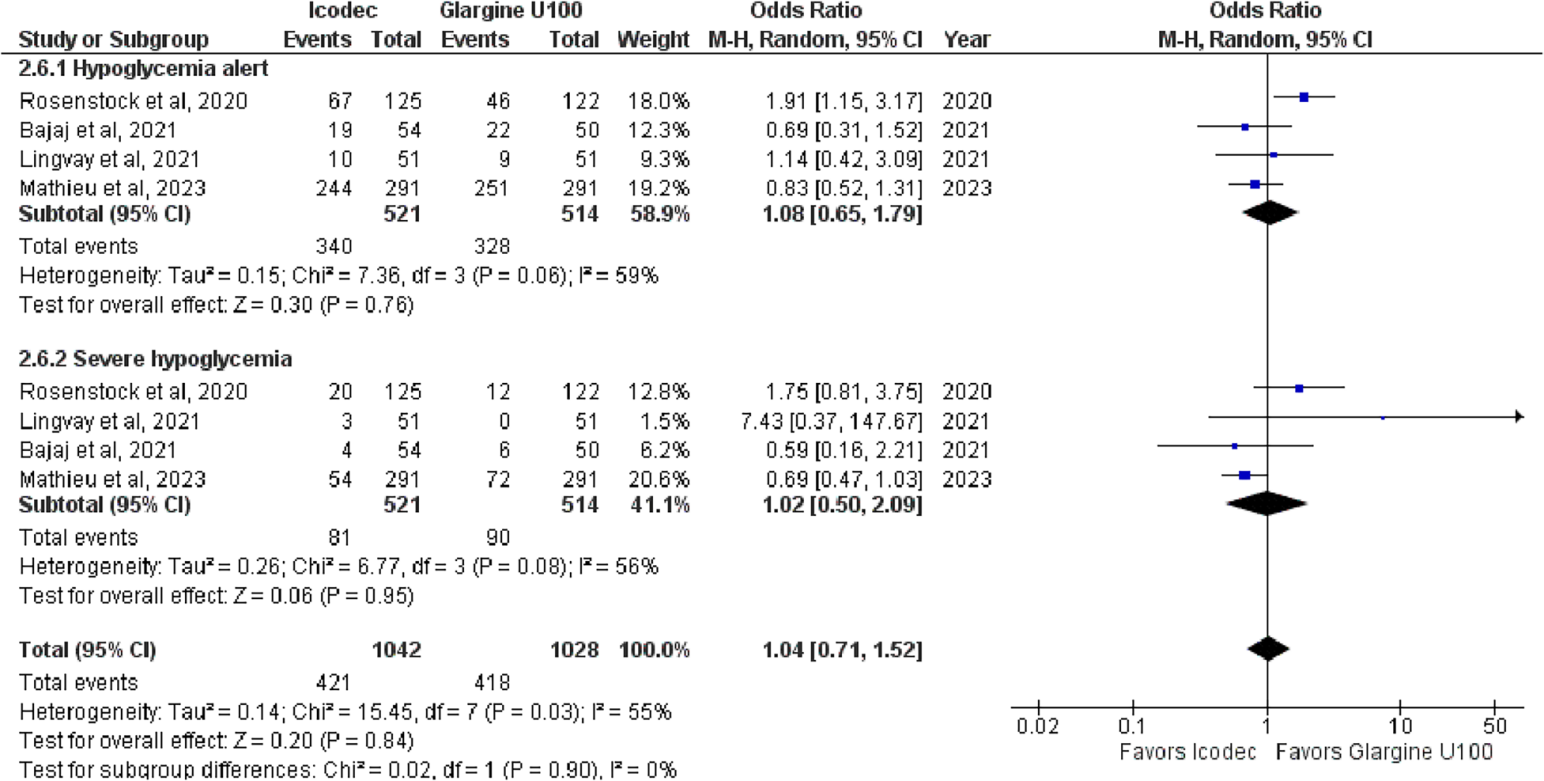
Forest plot of comparison: 1 Once weekly Insulin Icodec vs Once daily Insulin Glargine U-100, outcome: 1.6 Odds ratio of Overall Hypoglycemia. Subgroup analysis was performed based on the severity of hypoglycemia I.e., Hypoglycemia alert and combined clinically significant or severe hypoglycemia

The overall OR for hypoglycemia was 1.04 {95% CI (0.71, 1.52), p = 0.84, I^2^ = 55%}, (Fig. 8), indicating no significant difference between the two interventions. Subgroup analysis was performed based on the severity of hypoglycemia I.e., Hypoglycemia alert and combined clinically significant or severe hypoglycemia. The test for subgroup differences between Insulin Icodec and Insulin Glargine U-100 was insignificant (p-value = 0.90), (Fig. 8), demonstrating similar safety profiles in both subgroups.

**3. Estimated Mean Body Weight Change (kg)**

**Fig. 9:**
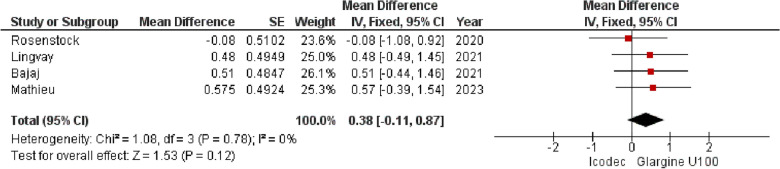
Forest plot of comparison: 1 Once weekly Insulin Icodec vs Once daily Insulin Glargine U-100, outcome: 1.7 Estimated Mean Change in Body weight (kg) from baseline

The overall MD in the estimated mean body weight change (kg) including all the four trials was 0.38 {95% CI (− 0.11, 0.87), p = 0.12, I^2^ = 0%}, indicating no significant difference between the two Insulin regimens, shown in (Fig. 9).

**4. Injection Site Reactions**

**Fig. 10:**
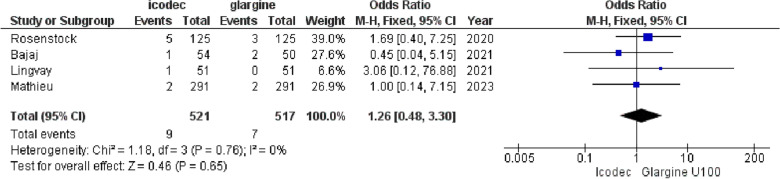
Forest plot of comparison: 1 Once weekly Insulin Icodec vs Once daily Insulin Glargine U-100, outcome: 1.8 Odds ratio for Injection site reactions

The OR for injection site reactions between Insulin Icodec versus Insulin Glargine U-100 was nonsignificant {OR = 1.26, (95% CI 0.48, 3.30), I^2^ = 0%, p = 0.65}, (Fig. 10).

**5. Hypersensitivity Reactions**

**Fig. 11:**
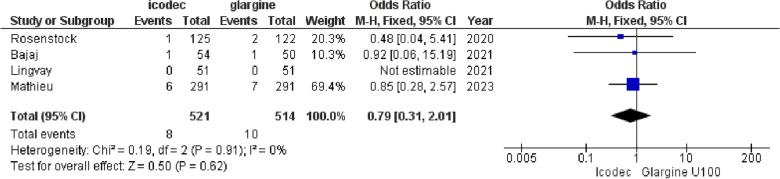
Forest plot of comparison: 1 Once weekly Insulin Icodec vs Once daily Insulin Glargine U-100, outcome: 1.9 Odds ratio for Hypersensitivity reactions

All the included studies reported incidence of hypersensitivity reactions among the two interventions, the OR for this comparison was found to be {OR = 0.79, (95% CI 0.31, 2.01), p = 0.62, I^2^ = 0%}, showing insignificant difference between the two drugs, (Fig. 11).

**6. Adverse Events Probably/Possibly Related to Basal Insulin**

**Fig. 12:**
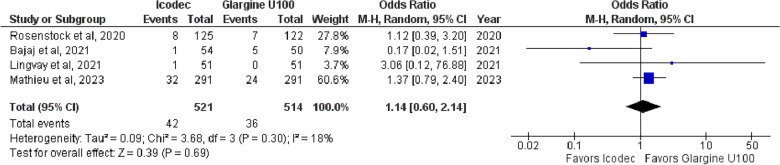
Forest plot of comparison: 1 Once weekly Insulin Icodec vs Once daily Insulin Glargine U-100, outcome: 1.10 Odds ratio for Adverse events probably/possibly due to basal insulin

The combined pooled analysis suggested no significant difference between Icodec and Once-Daily Insulin Glargine U-100 {OR = 1.14, 95% CI (0.60, 2.14), p = 0.69, I^2^ = 18%}, (Fig. 12).

## Discussion

This meta-analysis comprising of four studies with a patient population of 1035, aimed to comprehensively investigate the efficacy and safety of once-weekly Insulin Icodec compared to once-daily Insulin Glargine U-100 in individuals with T2D. This study showed that switching from daily Insulin Glargine U-100 to weekly Insulin Icodec showed longer TIR (%) as well as similar blood glycemic control and safety profile. Hence, it may be a good alternate option for management of longstanding T2D. In the past century, notable progress has been made in developing innovative Insulin formulations, including highly fast-acting and prolonged-release basal Insulin analogs. The latter is particularly vital for managing overnight fasting and keeping blood glucose levels within the normal physiological range during meals [21]. As mentioned by Bajaj et al. [22] basal Insulin is typically recommended for T2D when non-Insulin therapies prove insufficient to reach glycemic targets. Various obstacles associated with basal Insulin therapy for T2D contribute to the failure to achieve glycemic goals. These barriers include delays in initiating or adjusting Insulin, needle phobia leading to missed daily injections, instances of missed Insulin doses, Insulin discontinuation, and the occurrence of hypoglycemia [5]. A systematic review by Singh, Awadhesh Kumar et al. highlights Insulin Icodec as the most advanced insulin candidate suitable for once-weekly administration, showing potential in significantly reducing injection frequency by over 85% compared to once-daily basal insulin analogs [23] and offers a similar advantage as once-weekly compared to daily glucagon-like peptide-1 (GLP-1) receptor agonists [24].

Our pooled analysis reveals that patients receiving once-weekly Insulin Icodec experienced a 4.68% longer TIR compared to those on daily Insulin Glargine U-100. According to international consensus each 5% increase in TIR (%) is considered a clinically significant improvement in glycemic control [25]. Even though, our analysis represents a slightly lesser TIR (%) difference between the two drugs after the addition of the latest trial with a larger sample size, it may still show a better glycemic control with Insulin Icodec compared to Glargine U-100. Other efficacy outcomes such as the estimated mean change in HbA1c (%) and FPG (mg/dL) did not show significant differences between Insulin Icodec and Insulin Glargine U-100, consistent with the finding of previous meta-analyses by Ribeiro E Silva, Rodrigo et al., Abuelazm, Mohamed et al., and Shetty, Sahana, and Renuka Suvarna, all of which demonstrated decreased HbA1c, increased TIR, and similar hypoglycemic events [11, 26, 27]. Furthermore, the estimated mean alteration in HbA1c (%) from baseline was found to be similar between the two groups, which is a new finding as previous meta-analyses have found a significant improvement in glycated hemoglobin percentage with Insulin Icodec [11]. Notably, the odds ratio for patients achieving HbA1c < 7% did not exhibit a significant difference between the two treatments in our analysis, contrary to the findings of Shetty, Sahana, and Renuka Suvarna, which demonstrated that once-weekly insulin icodec achieved superior glycated hemoglobin reduction and a higher proportion of patients reaching HbA1c targets (< 7%) compared to daily basal insulin analogues [27]. The significantly higher efficacy of once-weekly insulin icodec compared to once- daily Insulin Glargine U-100 that it may be a preferred option for achieving excellent glycemic control in patients with type 2 diabetes.

Regarding safety outcomes, including estimated mean body weight change, overall hypoglycemia, adverse events related to Insulin, hypersensitivity, and injection site reactions, there were no significant differences in risk between the two Insulin regimens. These findings suggest that once-weekly dosing with Insulin Icodec may offer a convenient alternative to traditional daily injections without compromising glycemic control. This aligns with the conclusions drawn from previous network meta-analyses conducted by Wang, Peng et al., which underscored the superior glycemic control achieved by once-weekly insulin Icodec compared to insulin Fc in the context of type 2 diabetes [28].

The absence of notable differences in hypoglycemia episodes both hypoglycemia alerts and severe hypoglycemia was reassuring. The nonsignificant body weight changes between the two Insulin regimens are an additional advantage. This is in contrast to the findings reported by Abuelazm, Mohamed et al., where Once-weekly Insulin Icodec was associated with an increase in body weight [26]. Given that weight gain is a common apprehension with certain Insulin therapies, the observed similarity in this aspect presents a noteworthy advantage, potentially fostering greater compliance with the prescribed treatment regimen.

The associated heterogeneity was relieved by conducting a leave-one-out analysis and after removal of the outlier study, the heterogeneity concern was fully resolved. Importantly, the fact that the glycemic range target varied in between the included studies could have also impacted the overall findings.

. Leave-one-out analysis was also performed for the associated heterogeneity which got completely resolved after the removal of the *Matheiu *et al*.* [20] study. As mentioned by another recent meta-analysis [11] comparing Insulin Icodec with Daily Insulin analogues (Glargine U-100 and Degludec) lately, it has become standard to evaluate various Insulin analogues to determine whether one is superior or non-inferior to another by utilizing a “treat-to-target” approach, hence, it is expected for the glycemic control profile of both comparators to be similar. Despite some heterogeneity, the overall trend suggests comparable efficacy in achieving glycemic targets.

With that being said, the identification of comparable efficacy in long-term glycemic control between both interventions represents a significant breakthrough in diabetes management with multifaceted benefits. The reduced frequency of injections not only streamlines the treatment regimen but also alleviates the burden of daily administration, potentially improving patient adherence.

## Limitations

However, it's important to recognize some limitations in this meta-analysis. One significant limitation is the small number of trials considered, resulting in a relatively small overall group of participants. This could affect the reliability of the findings. Another issue is the varying durations of the included trials, which might make it harder to draw consistent conclusions. Hence, more long-term trials with larger sample sizes should be performed for further clarity. Lastly, in the study by *Bajaj *et al*.* [19] all the participants were already using basal Insulin therapy as opposed to the studies by *Lingvay *et al*.* [18] and *Rosenstock *et al*.* [17] which could have influenced the overall results as Insulin naive patients generally have a hard time adapting to the weekly dosage and are susceptible to hypoglycemic episodes in contrast to patients already using basal Insulin.

## Conclusions

In conclusions, our systematic review and meta-analysis showed that once-weekly Insulin Icodec exhibited more percentage time with glucose in range compared to once-daily Insulin Glargine U-100 and overall exhibited a comparable efficacy in glycemic control and a similar safety profile in patients with T2D. The identification of comparable efficacy in long-term glycemic control, coupled with the reduced injection frequency and reassuring safety profiles, marks a transformative development in the landscape of diabetes care. Thus, the implementation of this novel weekly Insulin regimen should be promoted in the management of T2D. Furthermore, additional well-designed studies are warranted to strengthen the validity of these findings.

### Supplementary Information

**Additional file 1: Table S1.** Reasons for exclusion of Excluded studies.

**Additional file 2: Figure S1.** Once weekly Insulin Icodec vs Once daily Insulin Glargine U-100, outcome: Leave-one-out analysis for Estimated Mean Change in Time with glucose in range (%) from baseline. Heterogeneity was resolved after removal of the outlier study by Mathieu et al. **Figure S2.** Once weekly Insulin Icodec vs Once daily Insulin Glargine U-100, outcome: Different estimation methods for in between study heterogeneity for Estimated Mean Change in Time with glucose in range (%) from baseline. **Figure S3.** Once weekly Insulin Icodec vs Once daily Insulin Glargine U-100, outcome: Leave-one-out analysis for Estimated Mean Change in HbA1c (%) from baseline. Heterogeneity was resolved after removal of the outlier study by Mathieu et al.

## Data Availability

The dataset supporting the conclusions of this article is included in this article.
