# Supplementary material for: Efficacy and safety of once-weekly insulin icodec compared to once-daily insulin g U-100 in patients with type II diabetes: a systematic review and meta-analysis
Source: Diabetol Metab Syndr. 2024 Apr 3;16:80. doi: 10.1186/s13098-024-01305-z (PMC10988795; doi:10.1186/s13098-024-01305-z)
Supplement: Supplementary file 1 — Additional file 1: Table S1. Reasons for exclusion of Excluded studies. [file 13098_2024_1305_MOESM1_ESM.docx]

**Reason for exclusion of excluded studies:**

| Study | Reason |
| --- | --- |
| 1. Pieber, Thomas R et al. | Cross over trials |
| 2. Plum-Mörschel, Leona et al. | Cross over trials |
| 3. Pieber, Thomas R et al. | No daily insulin glargine U100 for comparison |
| 4. Nishimura, Erica et al. | No daily insulin glargine U100 for comparison |

1. Pieber TR, Arfelt KN, Cailleteau R, et al. Hypoglycaemia frequency and physiological response after double or triple doses of once-weekly insulin icodec vs once-daily insulin glargine U100 in type 2 diabetes: a randomised crossover trial. Diabetologia. 2023;66(8):1413-1430.
2. Plum-Mörschel L, Andersen LR, Hansen S, et al. Pharmacokinetic and Pharmacodynamic Characteristics of Insulin Icodec After Subcutaneous Administration in the Thigh, Abdomen or Upper Arm in Individuals with Type 2 Diabetes Mellitus. Clin Drug Investig. 2023;43(2):119-127.
3. Pieber TR, Asong M, Fluhr G, et al. Pharmacokinetic and pharmacodynamic properties of once-weekly insulin icodec in individuals with type 2 diabetes. Diabetes Obes Metab. 2023;25(12):3716-3723.
4. Nishimura E, Pridal L, Glendorf T, et al. Molecular and pharmacological characterization of insulin icodec: a new basal insulin analog designed for once-weekly dosing. BMJ Open Diabetes Res Care. 2021;9(1):e002301.
