# Supplementary figures and images for: Efficacy and safety of once-weekly insulin icodec compared to once-daily insulin g U-100 in patients with type II diabetes: a systematic review and meta-analysis
Source: Diabetol Metab Syndr. 2024 Apr 3;16:80. doi: 10.1186/s13098-024-01305-z (PMC10988795; doi:10.1186/s13098-024-01305-z)

**Supp S1**

**
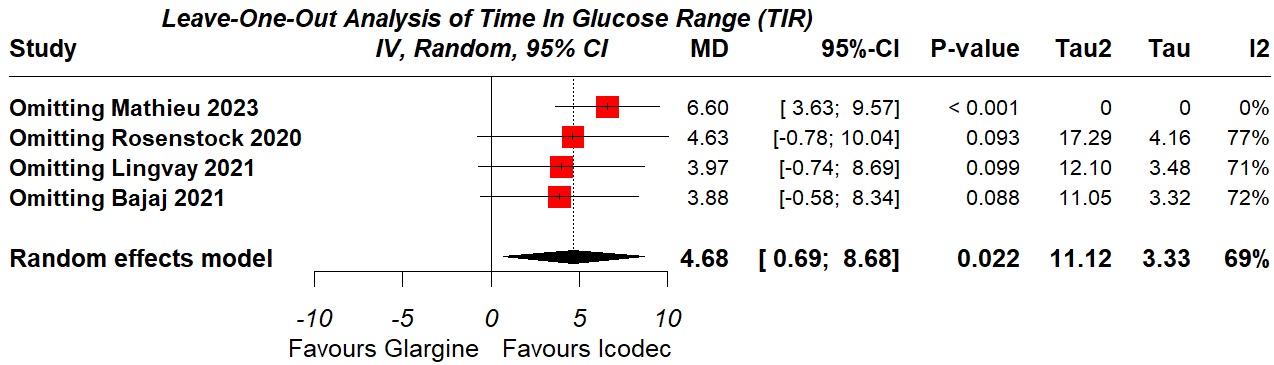
**

**Supp S2**

**
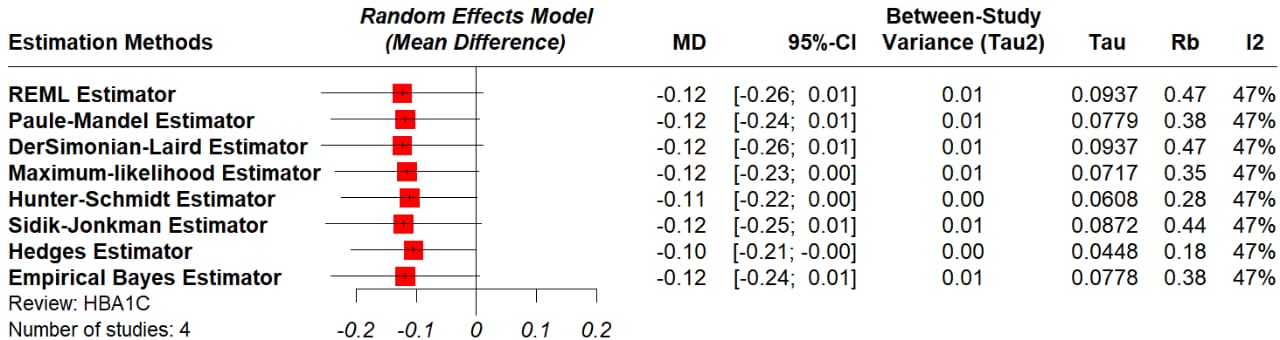
**

**Supp S3**

**
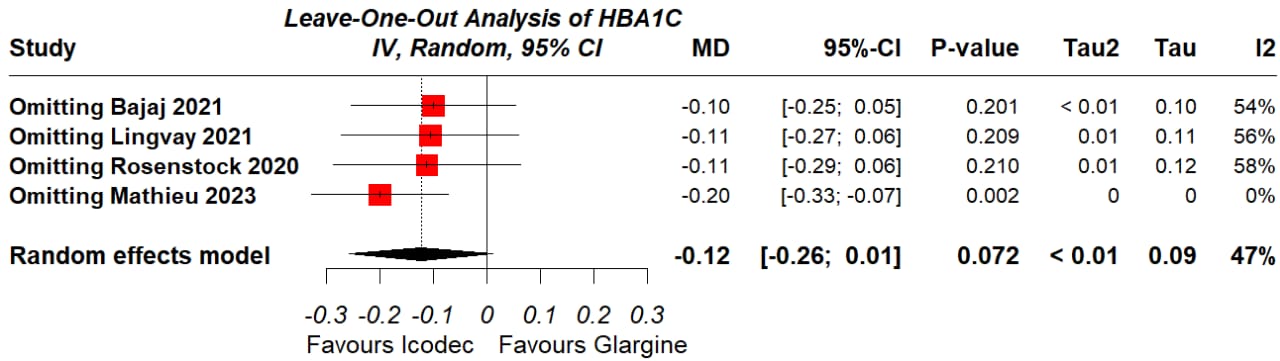
**

Supplement: Supplementary file 2 — Additional file 2: Figure S1. Once weekly Insulin Icodec vs Once daily Insulin Glargine U-100, outcome: Leave-one-out analysis for Estimated Mean Change in Time with glucose in range (%) from baseline. Heterogeneity was resolved after removal of the outlier study by Mathieu et al. Figure S2. Once weekly Insulin Icodec vs Once daily Insulin Glargine U-100, outcome: Different estimation methods for in between study heterogeneity for Estimated Mean Change in Time with glucose in range (%) from baseline. Figure S3. Once weekly Insulin Icodec vs Once daily Insulin Glargine U-100, outcome: Leave-one-out analysis for Estimated Mean Change in HbA1c (%) from baseline. Heterogeneity was resolved after removal of the outlier study by Mathieu et al. [file 13098_2024_1305_MOESM2_ESM.docx]
